# miR-338-5p-ZEB2 axis in Diagnostic, Therapeutic Predictive and Prognostic Value of Gastric Cancer

**DOI:** 10.7150/jca.58249

**Published:** 2021-09-23

**Authors:** Xiaoli Wei, Jiejie Zhu, Yiyin Zhang, Qihong Zhao, Hua Wang, Kangsheng Gu

**Affiliations:** 1 Department of Oncology, The First Affiliated Hospital of Anhui Medical University, Hefei, Anhui 230032, P.R. China.; 2 Department of Food and Nutrition Hygiene, School of Public Health, Anhui Medical University, Hefei, Anhui 230032, P.R. China

**Keywords:** Gastric cancer, miR-338-5p, ZEB2, cisplatin resistance

## Abstract

MiRNAs have been widely reported to be involved in the occurrence and development of cancers. So far, some studies have revealed that miR-338-5p has the functions of tumorigenesis and tumor suppression. However, the role of miR-338-5p in the pathogenesis, progression and treatment of gastric cancer (GC) has not been reported. MiRNAs microarray analysis showed for the first time that miR-338-5p was significantly lower-expression in cisplin-resistant GC cells SGC7901/DDP, and cell viability assay and flow cytometry confirmed that overexpression of miR-338-5p could significantly increase cisplatin-sensitivity of SGC7901/DDP and BGC823 cells. Subsequently, we found that the expression of miR-338-5p in postoperative cancer tissues of GC patients was also significantly lower than the corresponding paracancer tissues. The expression of miR-338-5p in peripheral blood serum of GC patients is generally lower than that of healthy people. Moreover, the low expression of miR-338-5p in the cancer tissues and serum of GC patients was closely associated with larger tumor volume, lymph node metastasis, later stage, and even poorer survival, which was confirmed by close 5-year cases follow-up. ZEB2, as a predictive target of miR-338-5p, its expression was negatively regulated by miR-338-5p and can promote cisplatin-resistance in SGC7901/DDP and BGC823 cells. The expression of ZEB2 in cisplatin-resistant SGC7901/DDP cells and GC tissues were significantly higher than SGC7901 cells and paracancer tissues, respectively. Moreover, the expression of ZEB2 in tumor tissues was negatively correlated with miR-338-5p in tumor tissues and peripheral blood serum of GC patients, and the abnormally high expression of ZEB2 in prospective case studies is positively related with more serious clinical pathology and worse survival. More meaningfully, in a retrospective case study, we found that high ZEB2 expression predicts worse clinical efficacy of platinum chemotherapy. Thus, miR-338-5p-ZEB2 axis have novel diagnostic, therapeutic predictive, and prognostic value in GC patients.

## Introduction

Gastric cancer (GC) is particularly prevalent among people worldwide. According to a new data, as many as 1000,000 cases were newly diagnosed with stomach cancer and 783,000 deaths occurred globally in 2018, making it the fifth most common diagnosed cancer and the third leading cause of cancer death 1. These research data suggest that it is crucially important to reveal the causes of GC. There are a wide variety of risk factors for GC such as tobacco, high salty and smoked food intake, decreasing servings of fruits and vegetables, Helicobacter pylori infection, male sex and family history, whereas taking nonsteroidal anti-inflammatory drugs (NSAIDs) and statins might diminish GC risk 2, 3. Most GCs occurs in the antrum and gastric body. Around 90 to 95 percent of all GCs are a type referred to as adenocarcinoma of the stomach. In this type, the cancer develops from the cells that form the mucosa, the most superficial lining of the stomach that produces mucus. A primary way of treatment for patients with GC is surgical resection, yet surgery cannot completely remove the lesion for the advanced cancer patients. Therefore, it requires chemotherapy to prolong its life survival. Fluorouracil and platinum are currently the most fundamental drugs for the treatment of GC 4.

Three platinum drugs (cisplatin, carboplatin, oxaliplatin) are applied to clinical treatment for cancer all over the world. Platinum drugs are cell cycle nonspecific agents (CCNSA). The mechanism of action is to enter the tumor cells and insert into DNA to form cross-links, block the production of DNA, mRNA and proteins, prevent DNA synthesis, manipulate signaling pathways which finally result in cell apoptosis or necrosis 5, 6. Although it is believed that platinum-based chemotherapy can extend survival time compared to best supportive care in advanced disease patients, it still remains a high rate of recurrence and metastasis due to resistance. Accumulating studies depicted that multiple mechanisms of cancer drug resistance, including: decreased drug uptake, drug sequestration, anti-apoptotic mechanisms, drug target proteins alteration, as well as enhanced DNA repair activity, etc. 7-9. The mechanisms of platinum drugs resistance in GC are still ambiguous.

MicroRNAs (miRNAs) are short 18-25 single-stranded noncoding RNAs. The first miRNA to be observed was lin-4 which negatively regulate LIN-14 translation in in the roundworm C. elegans in 1993 10. A wide variety of miRNAs diverse in structure, abundance, and expression profile are discovered since then. Functionally, miRNAs can direct genes expression, as part of a Ribonucleoprotein (RNP) Complex, providing a specific sequence to allow the RNP to act on a particular target 11. MicroRNAs reduce expression of their targets by RNA degradation 12, also inducing uridylation 13, inducing deadenylation 14, sequestration of the relevant mRNA 15, and other mechanisms. Aside from microRNAs, there are various noncoding RNAs (ncRNAs), including lncRNAs. The interplay of microRNAs and lncRNAs formats a network to regulate cellular function. MicroRNAs have been found to be related to numerous diseases like cancer. A great deal of studies illustrate that miRNAs are associated with tumor biological process and treatment respond. Overexpression of miR-193a-3p, miR-23a and miR-338-5p in both tissue and blood were detected in colorectal cancer 16. Viability and migratory ability increased in temozolomide resistant glioma cells overexpressing miRNA-27a-3p 17. This suggests that miRNAs may be used as biomarkers for early diagnosis, prognosis, and prediction 18-20.

Previous studies have demonstrated that miR-338-5p get involved in the regulation of tumorigenesis and tumor suppression. For example, Long J et al. found miR-338-5p enhances the proliferation and metastasis of malignant melanoma cell by targeting CD82 21. While Lei D et al. manifested that miR-338-5p play its tumor suppressor role via targeting EFEMP1 in glioma 22. In addition, miR-338-5p could also regulate chemoresistance of tumor cells to chemotherapeutic drugs. miR-338-5p sensitize hepatocellular carcinoma cells to doxorubicin (DOX) and vinblastine (VBL) through miR-338-5p/EGFR/ABCB1 regulatory loop 23. miR-338-5p reduce cell proliferation, colony formation, migration, and cisplatin resistance in esophageal squamous cancer cells by targeting FERMT2 24. Although a large number of studies have confirmed that miR-338-5p plays an important role in malignant tumors. However, up to now, studies on gastric cancer seem to be insufficient, because many previous studies mainly focused on the potential mechanism of miR-338-3p on various human gastric cancer cells 25-27, but failed to clarify miR-338-5p. miR-338-5p has only been reported to inhibit the growth of gastric cancer cells 28. There is a lack of effective research on the relationship between the expression of miR-338-5p in clinical gastric cancer samples and patients' clinicopathological characteristics, survival period. Therefore, miR-338-5p was carried out extensive research into various cancers, but rarely in GC.

Zinc finger E-box binding homeobox 2 (ZEB2), also named as SMAD-interacting protein-1 (SIP1), belongs to the ZEB family of several functional domains including two zinc-finger clusters separately. ZEB2 is a vital transcriptional regulator, which bind with E-box motif in promoters like E-Cadherin promoter and inhibite E-cadherin expression and downregulate other epithelial genes. Functionally, ZEB2 plays a major role in inducing the epithelialto-mesenchymal transition (EMT). Emerging data indicate that ZEB2 is closely related to EMT-induced processes such as development, differentiation, and malignant mechanisms, for example, cancer stem cell properties, apoptosis, cell cycle arrest, tumor growth, and metastasis 29-31. Beyond that, recent researches have shown that ZEB2 is involved in drug resistance in several types of tumors. Paired-box 6 (PAX6) can activate PI3K/AKT signaling pathway by directly binding the promoter region of ZEB2 in non-small cell lung cancer, thereby mediating cisplatin resistance 32. Transfection with ZEB2 siRNA in cisplatin-resistant SGC7901/DDP cells can enhance apoptosis and decrease in cell viability, making gastric cells sensitive to cisplatin *in vitro* 33. Briefly, ZEB2 is a critical regulator in cancer complexity. To our knowledge, no current evidence has been revealed with the hypothesis that miR-338-5p regulates GC cisplatin resistance by targeting ZEB2 and that an association between miR-338-5p and ZEB2 in clinical gastric cancer specimens.

To data, the expression of several miRNAs in tumors is significantly different from normal tissues and cells. The abnormal expression of these miRNAs is closely related to tumor cell invasion, metastasis and clinical prognosis. In our study, for the first time by comparing the differences in miRNA expression profiles between SGC7901/DDP cells and SGC7901 cells, we screened out miR-338-5p that is closely related to platinum resistance in GC cells and verified expression and function of miR-338-5p through *in vitro* experiments. And for the first time, it revealed the correlation between the expression of miR-338-5p in GC tissues and serum with clinicopathological data. More importantly, we conducted a prospective case study to study the correlation between the expression of miR-338-5p in tissues and serum with survival of GC patients. As a predicted target of miR-338-5p, the expression of ZEB2 was investigated to determine whether its expression was regulated by miR-338-5p and thus participated in GC cisplatin-resistance. The relationship between abnormal high expression of ZEB2 in GC tissues and clinicopathological parameters as well as survival prognosis of GC patients was analyzed through prospective case studies. More importantly, we collected clinical GC cases who received cisplatin or oxaliplatin based adjuvant chemotherapy and the patients were followed up, further explored the relationship between ZEB2 positive expression and the long-term clinical efficacy of platinum-containing chemotherapy in GC.

## Materials and methods

### Patients and tissue samples

We collected preoperative peripheral blood serum and postoperative GC tissue (tumor and para-tumor) samples from 50 patients with GC who underwent surgery in the First Affiliated Hospital of Anhui Medical University, Hefei, China from May to July 2015. There were 26 males and 24 females, with an average age of 53.5 years (ranging from 32 to 80 years) in **Table 1**. The inclusion criteria for patients enrolled in the study were as follows: i) The primary tumor tissues were confirmed to be gastric adenocarcinoma by histopathology; ii) No anticancer treatments including chemotherapy, radiotherapy, or targeted therapy were received before surgery; iii) Age between 18 and 80 years; iv) Physical condition score (ECOG score) ≤2 points 34; v) No serious underlying diseases (preoperative blood routine examination, liver and kidney function, ECG are generally normal); vi) complete clinical baseline data. The inclusion criteria were: i) Pregnant or lactating women; ii) Complicated with other serious diseases endangering life and health, such as developing second primary malignant tumors, uncontrolled diabetes, severe heart diseases, cerebral hemorrhage or cerebral infarction (within 6 months), chronic hepatic or renal failure, active tuberculosis and so on; iii) Patients with a history of severe mental disorders or central nervous system disorders; iv) Lost follow-up or incomplete follow-up information provided. Tissue samples and serum were immediately frozen in liquid nitrogen at the time of surgery, then stored at -80 °C until the extraction of RNA. Another group of tumor tissues was fixed with 10% formalin, embedded in paraffin and immunohistochemistry was performed. In addition, peripheral blood serums were collected from 30 healthy adults (healthy controls) registered in the First Hospital of Anhui Medical University, Hefei, China from Jun to Oct 2020 in **Table 1**. The healthy controls included in this study were all from physical examination centers and had no history of tumor, autoimmune disease or recent acute infection.

Also, the GC tissues (N=66) were collected from patients diagnosed as GC in the First Affiliated Hospital of Anhui Medical University from April 2013 to December 2018. The mean age of patients was 56.3 years (ranging from 37 to 72 years) in **Table 1**. In addition to meeting the above criteria, they all underwent radical R0 gastrectomy with D2 or more extensive lymphadenectomy for gastric cancer. Patients with distant metastasis of GC were excluded. And more than that, they all underwent 4 to 6 courses of adjuvant chemotherapy with cisplatin or oxaliplatin based drugs after surgery. The above GC patients all had complete clinical data.

There are 24 GC patients of the 50 were followed up successfully for 5 years from July 2015 to August 2020. Another group of 66 patients with gastric cancer all had successful follow-ups. They were followed up from May 2013 to October 2020. Disease-free survival (DFS) and Overall survival (OS) was recorded in these patients, according to the relapse and survival of patients from the beginning of enrollment to the end of follow-up.

### Cell culture

Human GC cell lines SGC7901 and BGC823 were obtained from the Cell Bank of the Chinese Academy of Sciences (Shanghai, China). The SGC7901/DDP cells with cisplatin resistance were purchased from KeyGen Biotech Co., Ltd. (Nanjing, China). Cells were maintained in RPMI-1640 medium containing 10% FBS, streptomycin (100 g/mL) and penicillin (100 U/mL) in a 37 °C incubator with 5% CO2. In order to maintain the cisplatin resistant phenotype, cisplatin (at a final concentration of 800 ng/mL) was added to the culture medium for the SGC7901/DDP cells. SGC7901/DDP cells were cultured for one week in medium without cisplatin before experimentation.

### RNA extraction

Total RNA was extracted from human GC tissue samples and cultured cells respectively using the TRIZOL reagent (Invitrogen, USA) strictly in accordance with the instructions for processing. A small amount of RNA solution was taken and its concentration and purity were detected on the spectrophotometer Nanodrop 2000 (Thermo Scientific, USA). When the A260/A280 ratio is between 1.8 and 2.0, the RNA purity is good. Write down each group of sample concentration, diluted quantitative RNA in each group with DEPC water. Dilute quantitative RNA with DEPC, and put it in -80 °C for use or long-term preservation.

### miRNA and mRNA microarray

Total RNA from GC cisplatin-resistant and cisplatin-sensitive cells were amplified and transcribed into fluorescent cDNA, and then the fluorescent labeled samples were hybridized to the Agilent miRNAs-mRNAs Human Gene Expression Microarray V4.0 (Capital Bio Corp, Beijing, China) following the manufacturer's recommendations. The microarray was scanned by an Agilent Microarray Scanner. Image processing was conducted using Agilent Feature Extraction software and raw microarray signals normalized using Agilent Gene-Spring software. The normalized miRNA and mRNA expression profiles data output was received in Excel spreadsheets. The two groups of samples data were analyzed by T-test to get the P-values. FC values represent the differently expressed mRNAs between SGC7901/DDP and their parental cells. Cluster 3.0 software was performed to show differential expression patterns of miRNAs and mRNAs.

### Real-time Polymerase Chain Reaction (RT-PCR)

RT-qPCR of miRNA was performed according to the manufacturer's instructions of Hairpin-it^TM^ microRNAs (GenePharma) and U6 snRNA Normalization RT-qPCR Quantitation kits (GenePharma). The primers sequences of miR-338-5p and U6 were as follows: miR-338-5p RT primer 5'-GTCGTATCCAGTGCAGGGTCCGAGGTATTCGCACTGGATACGACCACTCA-3', PCR primers, forward 5'-CGCGAACAATATCCTGGTGC-3' and reverse 5'-AGTGCAGGGTCCGAGGTATT-3'; and U6 RT primer 5'-CGCTTCACGAATTTGCGTGTCAT-3', PCR primers, forward 5'-GCTTCGGCAGCACATATACTAAAAT-3' and reverse 5'-CGCTTCACGAATTTGCGTGTCAT-3'. The expression level of miR-338-5p is expressed by 2 -ΔΔCt. ΔCt is the difference between the Ct value of the target gene and the reference gene (U6/β-actin) in the same sample.

### siRNA transfection and plasmid transfection of miRNA mimics

ZEB2-siRNA and plasmid of miR-338-5p mimics were purchased from Shanghai Gemma Pharmaceutical Technology Co., cell transfections were performed according to the instructions of Lipofectamine 2000 transfection reagent (Invitrogen, Carlsbad, CA). Cells were seeded in 2 mL of antibiotic-free medium the day before transfection. Diluting 80 pmol siRNA or 2 µg plasmid with 250 μl Opti-MEM™ (Reduced Serum Medium, Gibco, USA), followed by adding 5 µl of Lipofectamine 2000 into 250 µl Opti-MEM™ and incubate at room temperature for 5 minutes. The diluted siRNA or plasmid and Lipofectamine 2000 were mixed and stand at room temperature for 20 minutes. The mixture was then added to the culture dishes with cells. According to the experimental requirements, after 24-48 hours, relevant experimental operations were performed.

### Immunohistochemistry

For immunohistochemical (IHC) analysis, GC tumor tissues were fixed with 10% formalin, embedded in paraffin. The wax block was sliced to a thickness of 4 µm, infiltrated with xylene and soaked in graded concentrations of ethanol and incubated with 3% H2O2 deionized water to block endogenous peroxidase. Then, the sections were incubated with primary antibody overnight at 4 °C. After rinsed with PBS, further add goat anti-rabbit IgG antibody-HRP polymer to the slide. Finally observe staining using diaminobenzidine (DAB) and hematoxylin successively under a microscope. We selected 3 fields of view of the slice not less than 200 tumor cells under high magnification randomly. Determination of ZEB2 staining results: the positive signal of ZEB2 protein was mainly located in the cytoplasm and showed brown-yellow particle-like substances. According to the percentage of positive cells and color intensity, the results were judged. A histochemical score (H-score) was used to estimate protein expression. (1) The score of positive cell percentage : <5% is 0 score, 5-25% is 1 score, 26-50% is 2 score, and >50% is 3 score. (2) The score of color intensity: 0 score for no color, 1 score for pale yellow, 2 score for brown yellow and 3 score for dark brown. ZEB2 expression was assessed by multiplying scores representing the percentage of positive cells and color intensity. Scores of >4 was defined as positive, whereas <4 means negative. Each slide was viewed and scored by at least two seasoned pathologists independently.

### Western blotting

Protein was extracted by lysis and centrifugation. The protein content was quantified according to the BCA kit instructions. Protein lysates were further loaded onto prepared PAGE gels, transferred to polyvinylidene fluoride (PVDF) membrane after electrophoresis. The membrane was placed in blocking solution and incubatd with diluted primary and secenondary antibodies. The membrane was washed with TBST for 3×10 min, ending up with protein signal detection on the visualization instrument. The antibody of ZEB2 and β-actin were purchased from Abcam company. Tween-20 is purchased from Sigma; TBS were purchased from boshide company; Protein marker Maker was purchased from Beijing zhongshan jinqiao biotechnology co., LTD.

### Cell viability assay

SGC7901/DDP and BGC823 cells were seeded on a 96-well plate. After 24 h, SGC7901/DDP cells were treated with cisplatin at 40, 20, 2, 0.2, 0.02 and 0.002 µg/mL for 48 hours. 20 µl MTT (5 mg/ml; Sigma-Aldrich; Merck KGaA) was added to each well. After incubation for 4 h, 150 µl DMSO (Sigma-Aldrich; Merck KGaA) was added and gently shake well for 10 minutes. The absorbance at 490 nm was determined by an ELx800 spectrophotometer (BioTek Instruments, Inc.). The median inhibitory concentration (IC50) of cisplatin was calculated according to the cell activity by SPSS 16.0 software. All reactions were repeated three times.

### Apoptosis assay

The apoptotic rates of GC cells were evaluated by flow cytometry (FCM) with an Annexin V-FITC/propidium iodide (PI) Cell Apoptosis Detection kit (BestBio). About 1×10^6^ cells were treated with cisplatin at a final concentration of 1 µg/ml, respectively. Following 48 hours of treatment at 37 °C, the cells were collected and washed twice with ice-cold PBS. Cells were resuspended with 400 µl of 1X binding buffer and maintained at a final density of approximately 1×10^6^ cells/ml. Annexin V-FITC (5 µl) was added to the suspension, which was incubated in the dark for 15 minutes at room temperature. Following the addition of PI (10 µl) to the suspension, cells were incubated for an additional 5 minutes in the dark at room temperature. Subsequently, cell apoptosis was assessed using a Gallios flow cytometer (Beckman Coulter, Inc.).

### Statistical Analysis

The data were analysed with SPSS 19.0 software (IBM Corporation). Bands of western blots were quantified as gray values with the Tanon gel imaging system.The data were presented as mean ± standard deviation (mean ± SD). The Student's t-test (two groups) or one-way analysis of variance (ANOVA, more than two groups) was used to identify the differences among variables. The Chi-squared test was done to analyse the Immunohistochemical results. The comparison between two groups on the differences of miR-338-5p expression levels among GC patients was analyzed by the the Wilcoxon-Mann-Whitney test with the Bonferroni adjustment. The Kaplan-Meier survival curves were constructed to estimate the cumulative survival. The log-rank test was done to compare the survival rate between the two groups. *P*-value <0.05 was defined as statistically significant. Data was plotted using the Graphpad software.

## Results

### Decreased expression of miR-338-5p in GC cells

**Figure 1:**
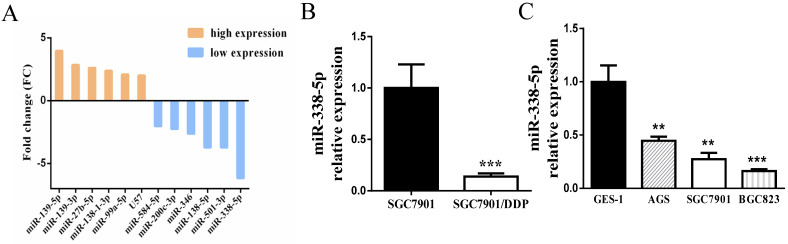
** Decreased expression of of miR-338-5p in GC cells. (A)** Microarray assay of miRNA expression levels between cisplatin-resistant SGC7901/DDP cells and cisplatin-sensitive SGC7901 cells. The graphic shows only the upregulated and downregulated miRNAs as defined by a P-value < 0.05 and a FC > 2-fold difference in expression relative to the control cells. The red mark indicates miR-338-5p. **(B)** qRT-PCR analysis showing the expression levels of miR-338-5p in the SGC7901/DDP and SGC7901 cells, *** *P*<0.001. **(C)** qRT-PCR analysis showing the expression levels of miR-338-5p in the GES-1, AGS, SGC7901 and BGC823 cells, ** *P*<0.001 and *** *P*<0.001.

Initially, the sensitivity of the cisplatin-resistance cell line SGC7901/DDP and its parental cell line SGC7901 in this experiment has been detected by MTT assay in the previous period, confirming that the cisplatin resistance of SGC7901/DDP cells is significantly greater than that of SGC7901 cells 35. The differential miRNA expression profiles between SGC7901/DDP cells and parental SGC7901 cells were obtained by miRNA microarray analysis 36. The Affymetrix miRNA GeneChip 4.0 was used to scan and quantify the signal intensity of probes of 1,316 human mature miRNAs on the chips for the 2 cell lines. The results manifested that 41 miRNAs were obviously differentially expressed (by *P*-value < 0.05 and FC > 2-fold) in SGC7901/DDP cells relative to the parental cells, including 12 upregulated miRNAs and 29 downregulated miRNAs. As shown in **Fig. 1A**, the most prominent expressed difference was miR-338-5p, which was revealed to be markedly downregulated in SGC7901/DDP compared with SGC7901 cells. RT-PCR also confirmed that miR-338-5p was severely down-regulated in SGC7901/DDP cells (**Fig. 1B**, *P*<0.001). Compared with human gastric normal mucosal epithelial cells GES-1, miR-338-5p is generally lower-expressed in various gastric cancer cell lines (**Fig. 1C**, ** *P*<0.001 and *** *P*<0.001). The datas indicate that miR-338-5p may be involved in the formation of cisplatin resistance in GC. Therefore, miR-338-5p was selected for further research as its function in GC remains unknown.

### Upregulation of miR-338-5p alleviated cisplatin resistance in GC cells

**Figure 2:**
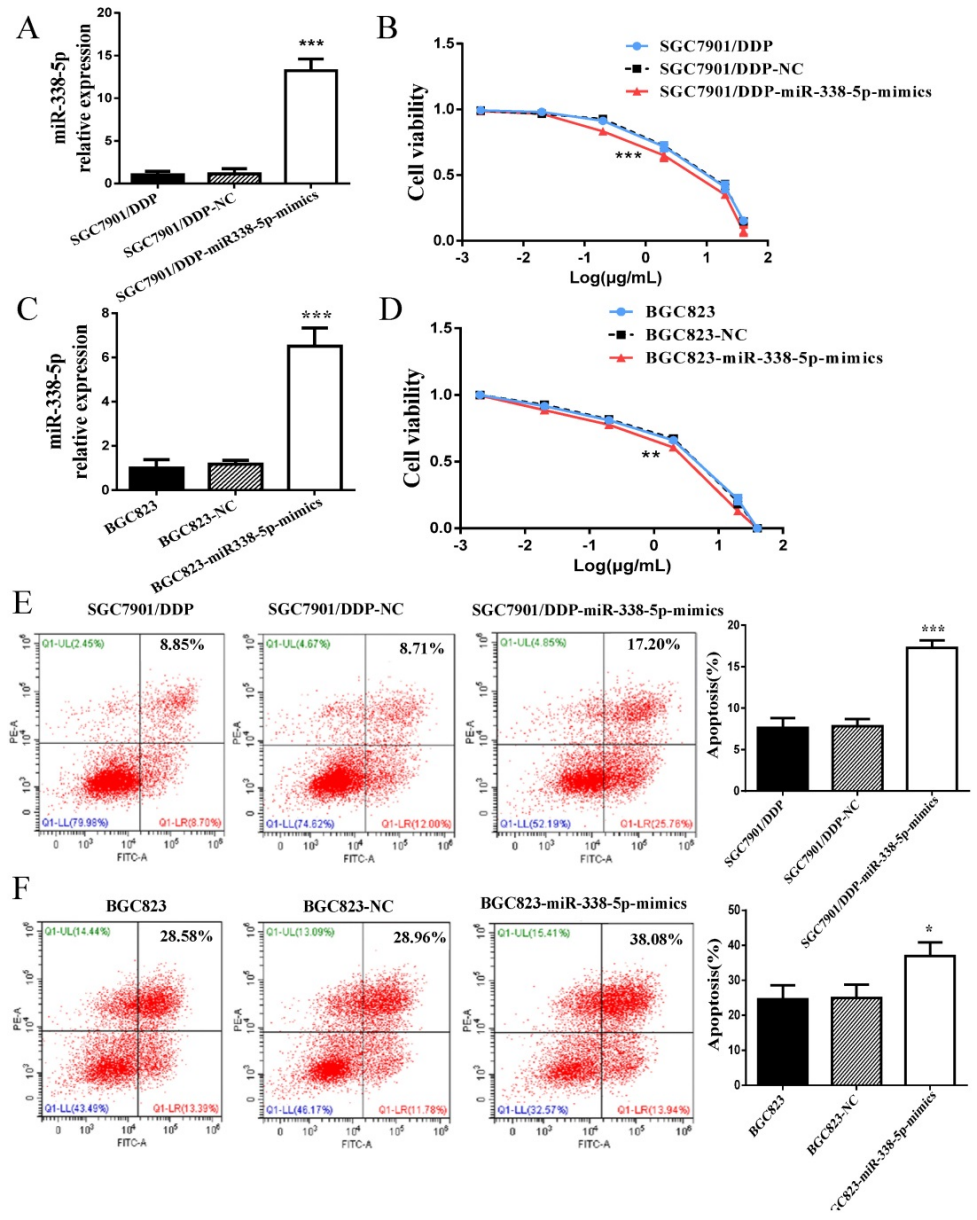
** Upregulation of miR-338 -5p alleviated cisplatin resistance in GC cells. (A and C)** qRT-PCR analysis showing the expression levels of miR-338-5p in the SGC7901/DDP and BGC823. *** *P*<0.001. **(B and D)** The cell viability of SGC7901/DDP and BGC823 cells after 48 h treatment with cisplatin at 40, 20, 2, 0.2, 0.02 and 0.002 g/mL. ***P<0.001. **(E and F)** Flow cytometry analysis showing apoptotic rate after 48h cisplatin treatment (final concentration 1 µg/ml) in SGC7901/DDP and BGC823 cells. ***P<0.001, *P<0.05. SGC7901/DDP-NC, SGC7901/DDP cells transfected with negative control; SGC7901/DDP-miR-338-5p-mimics, SGC7901/DDP cells transfected with microRNA-338-5p mimisc. BGC823-NC, BGC823 cells transfected with negative control; BGC823-miR-338-5p-mimics, BGC823 cells transfected with microRNA-338-5p mimisc.

In view of the abnormally low expression of miR-338-5p in GC cisplatin-resistant cells, we overexpressed miR-338-5p in SGC7901/DDP cells by plasmid transfection to explore whether miR-338-5p is involved cisplatin-resistance of GC cells. The expression level of miR-338-5p in SGC7901/DDP cells transfected with miR-338-5p-mimics plasmid was significantly higher than that in SGC7901/DDP and SGC7901/DDP- negative control (NC) cells (**Fig. 2A**), which was confirmed by RT-PCR. Subsequently, SGC7901/DDP cells were treated with different concentrations of cisplatin for 48 hours. The IC50 for cisplatin was estimated based on cell viability. Results showed that the miR-338-5p mimic cells exhibited a poor survival status compared with control SGC7901/DDP-NC and SGC7901/DDP cells (**Fig. 2B; Table 2**). Following 48 h treatment with cisplatin at a final concentration of 1 µg/ml, the SGC7901/DDP cells over-expression miR-338-5p exhibited significantly higher apoptotic rates compared with the control cells (**Fig. 2E**). Similarly, higher-expression of miR-338-5p in BGC823 GC cells also enhanced the sensitivity of cells to cisplatin, which can be confirmed by decreased cell viability and increased apoptosis (**Fig. 2C-D and 2F**). Data indicated that upregulation of miR-338-5p may enhance the cisplatin sensitivity of GC cells.

### Decreased expression of miR-338-5p in GC specimens

**Figure 3:**
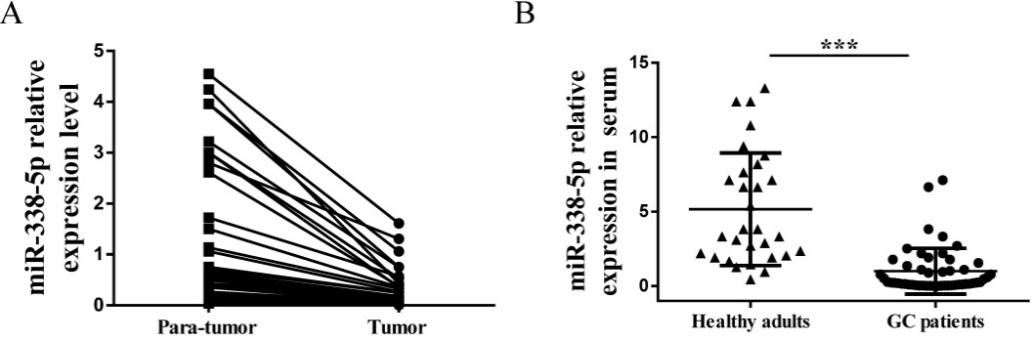
** Expression of miR-338-5p in tissues and serum of patients with gastric cancer. (A)** qRT-PCR analysis showing the expression levels of miR-338-5p in the tumor tissues and para-tumor tissues for 50 GC patients. *P*<0.001. **(B)** qRT-PCR analysis showing the expression levels of miR-338-5p in the Peripheral blood serum for 50 GC patients and 30 healthy adults. ***P<0.001.

Because of this interesting differential expression of miRNA-388-5p in GC cisplatin-resistant cells, we have the motivation to further analyze its expression in clinical GC specimens. We collected preoperative peripheral blood serum and postoperative GC tissues (tumor and para-tumor) samples from 50 patients. There were 26 males and 24 females, with an average age of 53.5 years (ranging from 32 to 80 years). The number of cases with tumor size less than 5cm was 35, and the number of cases with more than 5cm was 15. There were 28 cases of moderately differentiated and well-differentiated tumors, and there were 22 cases of poorly differentiated and undifferentiated tumors. The number of cases with lymph node metastasis was 30, and the number of cases without lymph node metastasis was 20. There were 38 patients at TNM I/II stage, and 12 patients at TNM III/IV stage. Expression of miR-338-5p in tissues and peripheral blood serum was tested by qRT-PCR. GC tumor tissues showed a significantly downregulated miR-338-5p expression compared with the para-tumor tissues (**Fig. 3A**,* P*<0.001). Meanwhile, the expression of miR-338-5p in peripheral blood serum of GC patients was generally lower than that of healthy adults, which was confirmed by comparison with peripheral blood serum of 30 healthy adults (**Fig. 3B**,* P*<0.001).

Furthermore, we analyzed the correlation between the expression of miR-338-5p in tumor tissues and peripheral blood serum with the pathological characteristics of GC patients. As shown in **Table 3**, the expression of miR-338-5p in tumor more than 5 cm was lower than that in tumor less than 5 cm (*P*<0.01). Patients with lymph node metastasis were associated with a marked lower expression level of miR-338-5p on average (*P*<0.001). In addition, patients at TNM III/IV stage had significantly lower miR-338-5p expression compared with patients at TNM I/II stage (*P*<0.05). No significant correlation was found between miR-338-5p expression and age, gender, differentiation (both *P*>0.05). Therefore, the above data indicate that miR-338-5p may act as a tumor-suppressor role in GC.

### Upregulated expression of ZEB2 in GC cisplatin-resistant cells

**Figure 4:**
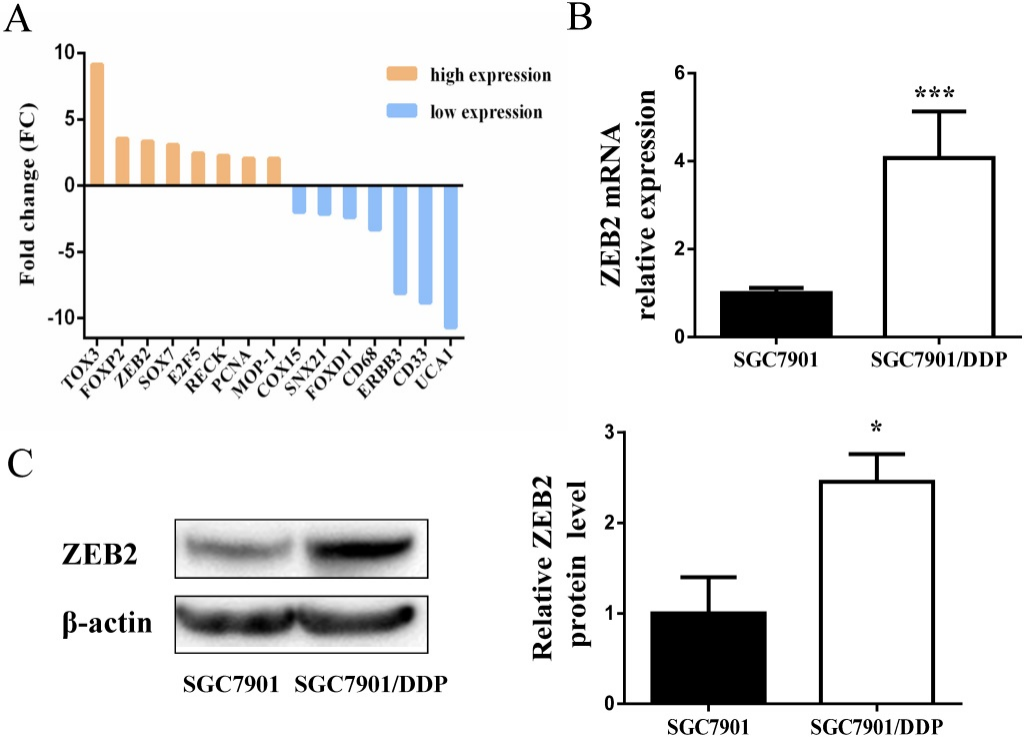
** Expression levels of ZEB2 in SGC7901/DDP cells were obviously higher than those in the SGC7901 cells. (A)** Microarray assay of mRNA expression levels between cisplatin-resistant SGC7901/DDP cells and cisplatin-sensitive SGC7901 cells. The graphic shows only the upregulated and downregulated mRNAs as defined by a *P-*value < 0.05 and a FC > 2-fold difference in expression relative to the control cells. The red mark indicates ZEB2 mRNA. **(B)** qRT-PCR analysis showing the expression levels of ZEB2 mRNA in the SGC7901/DDP and SGC7901 cells, *** *P*<0.001. **(C)** Western blot showing the ZEB2 protein levels in the SGC7901/DDP and SGC7901 cells using β-actin as a loading control, *P<0.05.

Subsequently, DIANAmT, miRanda, miRDB, miRWalk, RNAhybrid, PICTAR4, PICTAR5, PITA, RNA22 and Targetscan databases were used to search for potential targets of miR-338-5p (**Table 4**). ZEB2 is predicted to be one of the targets of miR-338-5p. In the previous experiment, we also conducted mRNA microarray chip detection between SGC7901/DDP cells and parental SGC7901 cells 35. As shown in **Fig. 4A**, the mRNA expression of ZEB2 in SGC7901/DDP cells was higher than that in SGC7901 cells. Interestingly, correlation analysis of miRNA and mRNA microarray showed that miR-338-5p was closely related to ZEB2 expression in SGC7901 and SGC7901/DDP cells (**Table 5**). Ulteriorly, the expression of ZEB2 mRNA was detected by qRT-PCR were consistent with the microarray results (**Fig. 4B**). In addition, the protein expression levels of ZEB2 in SGC7901/DDP cells were obviously higher than those in the SGC7901 cells based on western blot analysis (**Fig. 4C**). Hence, these results indicate that there may be a certain correlation between miR-338-5p and ZEB2 in cisplatin resistance of GC.

### ZEB2 is negatively regulated by miR-338-5p

**Figure 5:**
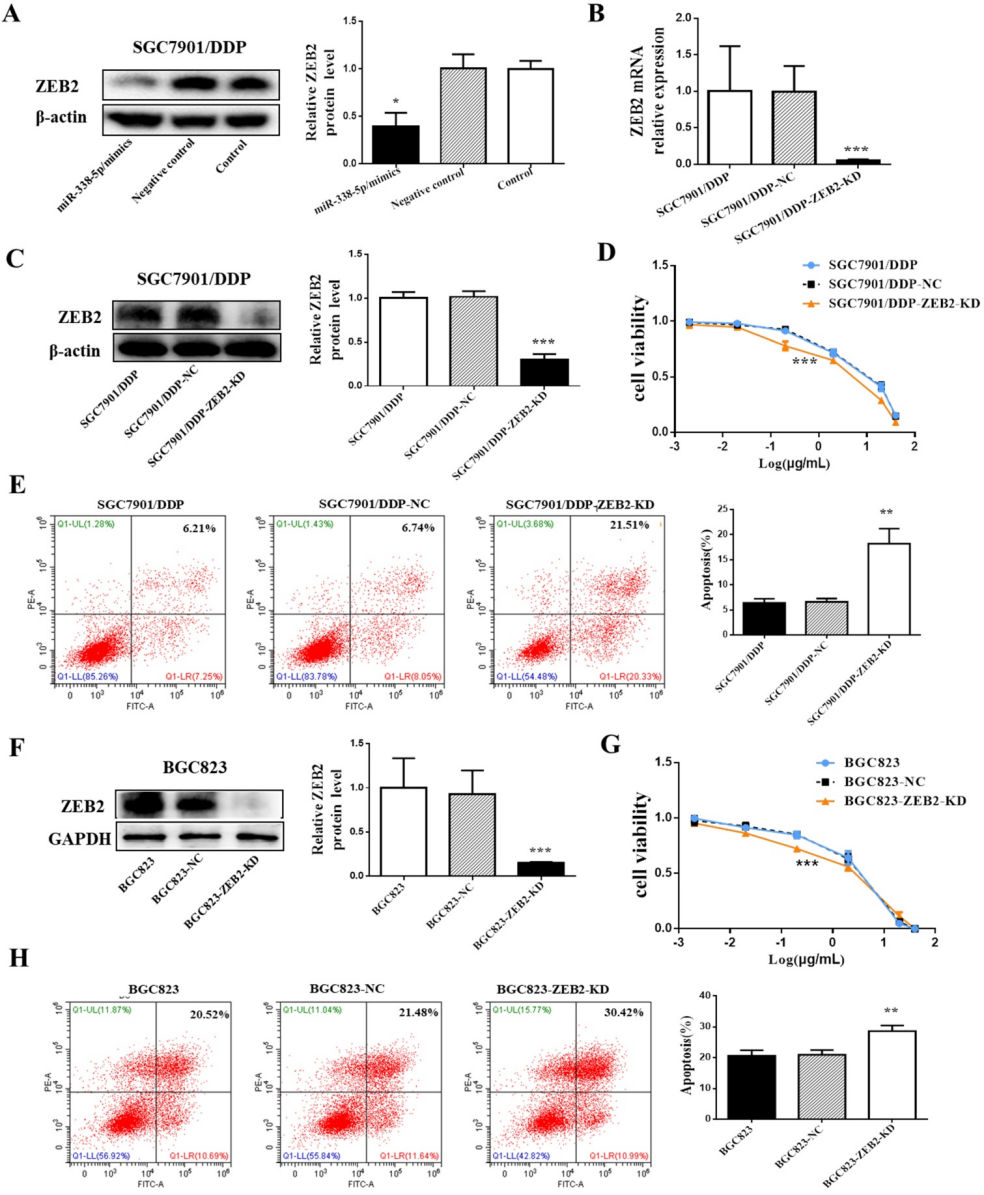
** Decreased expression of ZEB2 alleviated cisplatin resistance in GC cells. (A)** Western blot showing the ZEB2 protein levels in the SGC7901/DDP cells using β-actin as a loading control, *P<0.05. NC, Negative control, SGC7901/DDP cells transfected with Negative control. miR-338-5p mimics, SGC7901/DDP cells transfected with microRNA-338-5p mimics. Control, SGC7901/DDP cells without any treatment. **(B)** qRT-PCR analysis showing the expression levels of ZEB2 mRNA in the SGC7901/DDP. ***P<0.001. **(C and F)** Western blot showing the ZEB2 protein level in the SGC7901/DDP and BGC823 cells using β-actin or GAPDH as a loading contro, respectivelyl. *P<0.05. **(D and G)** The cell viability of SGC7901/DDP cells after 48 h treatment with cisplatin at 40, 20, 2, 0.2, 0.02 and 0.002 g/mL. ***P<0.001. (E and H) Flow cytometry analysis showing apoptotic rate after 48h cisplatin treatment (final concentration 1 µg/ml) in SGC7901/DDP and BGC823 cells, respectively.**P<0.01. SGC7901/DDP-NC, SGC7901/DDP cells transfected with negative contro; SGC7901/DDP-ZEB2-KD, SGC7901/DDP cells transfected with ZEB2-siRNA; BGC823-NC, BGC823 cells transfected with negative control; BGC823-ZEB2-KD, BGC823 cells transfected with ZEB2-siRNA.

For the purpose of clarifying the potential relationship between miR-338-5p and ZEB2, We transfected miR338-5p/mimics into SGC7901/DDP cells which originally has a lower expression of miR-338-5p. Results of qRT-PCR predictably confirmed that the expression of miR-338-5p in SGC7901/DDP cells transfected with miR338-5p/mimics was obviously higher than those in SGC7901/DDP cells transfected with negative control (**Fig. 2A**). It's worth noting that the SGC7901/DDP cells transfected with miR338-5p/mimics had significantly lower protein expression of ZEB2 by comparison with that in the control group (**Fig. 5A**). Collectively, the results of the present study demonstrated that ZEB2 is negatively regulated by miR-338-5p in GC cisplatin-resistant cells.

### Decreased expression of ZEB2 alleviated cisplatin resistance in GC cells

To investigate the role of ZEB2 in cisplatin resistance in GC, ZEB2 protein expression in SGC7901/DDP cells was knocked down by ZEB2-siRNA. After verifying the knockdown efficiency by Western blotting and qRT-PCR (**Fig. 5B and 5C**), SGC7901/DDP cells were then treated with cisplatin of different concentrations for 48 hours. Finally, the sensitivity of SGC7901/DDP cells to cisplatin was significantly increased after ZEB2 depletion (**Fig. 5D**). Following 48h treatment with cisplatin at a final concentration of 1 µg/ml, the SGC7901/DDP cells lower-expression ZEB2 exhibited significantly higher apoptotic rates compared with the control cells (**Fig. 5E**). Similarly, knockdown of ZEB2 expression in BGC823 GC cells also enhanced the sensitivity of cells to cisplatin, which can be confirmed by decreased cell apoptosis and increased viability (**Fig. 5F-H**). As a result, ZEB2 may be involved in cisplatin resistance of GC cells as a target of miR-338-5p.

### Expression levels of ZEB2 in GC specimens

**Figure 6:**
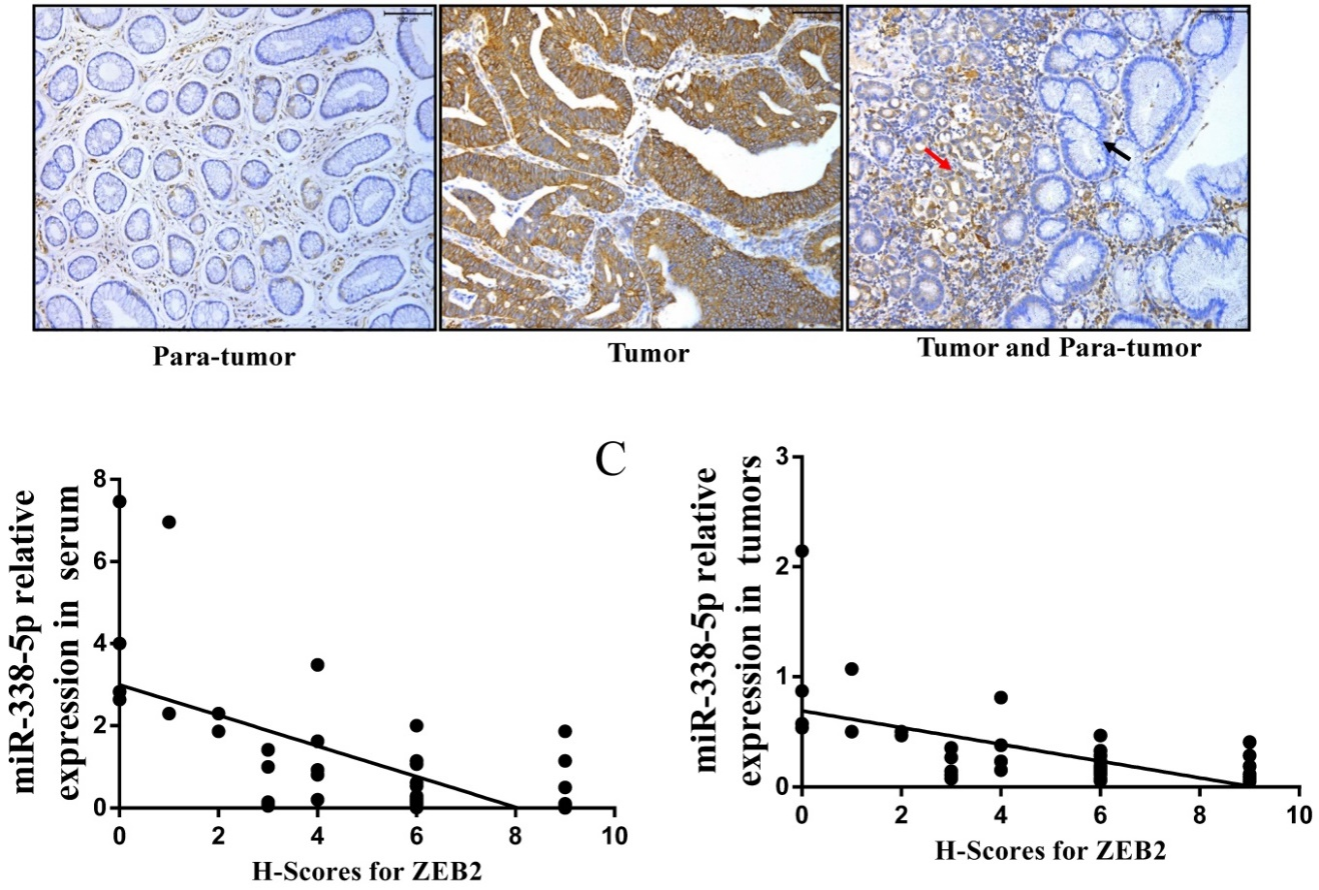
** The correlation between the H-scores for ZEB2 in tumor tissues and the expression of miR-338-5p in tissues and peripheral blood serum of GC patients. (A)** ZEB2 expression in postoperative tumor tissues and para-tumor tissues of 50 GC patients were detected using immunohistochemistry. **(B)** Correlation analysis between miR-338-5p expression in peripheral blood serum and ZEB2 expression in postoperative tumor tissues of 50 GC patients. r = - 0.6342, P < 0.001. **(C)** Correlation analysis between miR-338-5p expression and ZEB2 expression in postoperative tumor tissues of 50 GC patients. r = - 0.6007, *P* < 0.001. H-score, Histochemical score.

In view of the above-mentioned ZEB2 expression in GC cells and its correlation with miR-338-5p, we further analyzed the expression of ZEB2 via immunochemistry in the above 50 clinical GC specimens. Positive ZEB2 expression in tumor tissues was more frequent in para-tumor (**Fig. 6A and Table 6**). The number of cases with ZEB2 positive expression in tumor tissues was 36. In contrast, ZEB2 positive expression in para-tumor was only 5. In particular, as shown in the **Fig. 6A**, the expression of ZEB2 is gradually increased from paracancer to cancer tissues. Furthermore, we analyzed the correlation between the expression of ZEB2 and the pathological characteristics of GC patients. As shown in **Table 7**, patients with large volume of tumor, lymph node metastasis and late TNM stage were associated with a marked higher expression level of ZEB2 on average (*P*<0.01). No significant correlation was found between ZEB2 expression and gender, age, differentiation (both *P*>0.05). Interestingly, as shown in **Table 3**, the expression of miR-338-5p in tumor tissues with ZEB2 positive expression was significantly lower than that in tumor tissues with ZEB2 negative expression. There is a statistically significant correlation between the H-scores for ZEB2 in tumor tissues and the expression of miR-338-5p in tissues and peripheral blood serum of GC patients (**Fig. 6B**, r = - 0.6342, *P* < 0.001; **Fig. 6C**, r = - 0.6007, *P* < 0.001). In summary, ZEB2 is highly expressed in GC tissues and negatively correlated with the expression of miR-338-5p.

### The correlation between miR-338-5p/ ZEB2 expression and the survival rate in patients with gastric cancer

**Figure 7:**
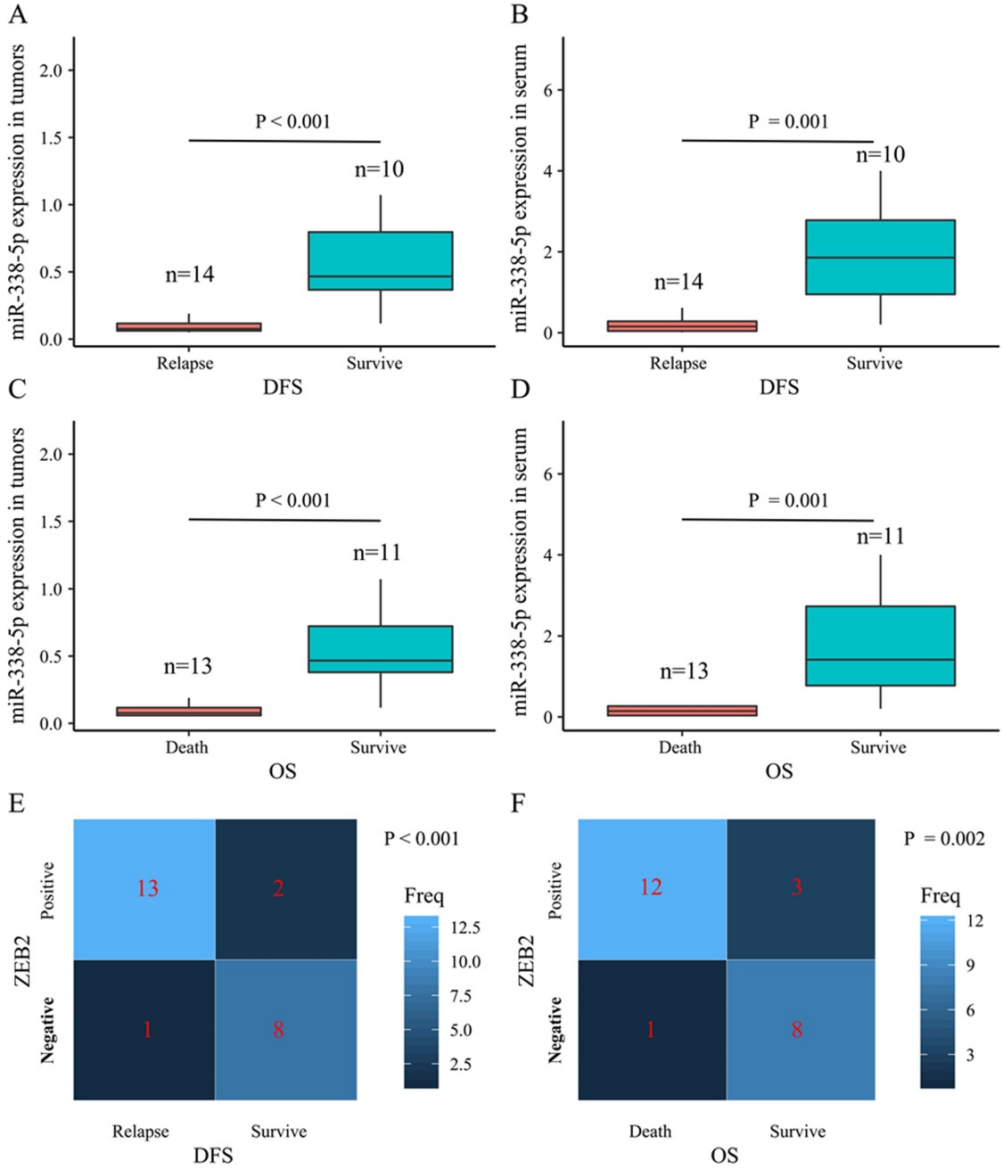
** The relationship between levels of miR-338-5p, ZEB2 expression and the survival rate in 24 GC patients. (A)** The poor prognosis for DFS was correlated with the low expression level of miR-338-5p in the tumor tissues, P<0.001. **(B)** The poor prognosis for DFS was correlated with the low expression level of miR-338-5p in the Peripheral blood serum, P=0.001. **(C)** The poor prognosis for OS was correlated with the low expression level of miR-338-5p in the tumor tissues, P<0.001. **(D)** The poor prognosis for OS was correlated with the low expression level of miR-338-5p in the Peripheral blood serum, P=0.001. **(E)** The relationship between ZEB2 expression and DFS, P<0.001. **(F)** The relationship between ZEB2 expression and OS. P=0.002.The l ines within the boxes represent the median values, and the edges of the boxes demonstrate the interquartile ranges. The lines outside the boxes demonstrate the 95% confidence intervals. GC, gastric cancer; OS, overall survival; DFS, disease-free survival.

The present study performed a five-year follow-up successfully for 24 patients with gastric cancer. The Wilcoxon-Mann-Whitney test with the Bonferroni adjustment was performed for comparisons on the expression level of miR-338-5p between groups. The ZEB2 expressions for DFS and OS rates at 5 years were calculated using the χ^2^ test. The box plots showed that the expression levels of miR-338-5p in the tumor tissues for GC patients survived after 5 years were higher than that for patients suffering relapse or death (**Fig. 7A** and **7C**), which were consistent with the expression level of miR-338-5p in peripheral blood serum (**Fig. 7B** and **7D**). The results revealed that patients with ZEB2 negative could achieve better DFS and OS, while patients with positive expression of ZEB2 exhibited a shorter survival time (**Fig. 7E**-**F**). Statistical analyses demonstrated that miR-338-5p and ZEB2 expression were both significantly associated with the DFS and OS at 5 years of GC patients. It suggested that GC patients with high miR-338-5p expression and negative expression of ZEB2 related to a better 5-year survival rate.

### Association between ZEB2 expression and the prognostic efficacy in patients with gastric cancer who received cisplatin or oxaliplatin based adjuvant chemotherapy

**Figure 8:**
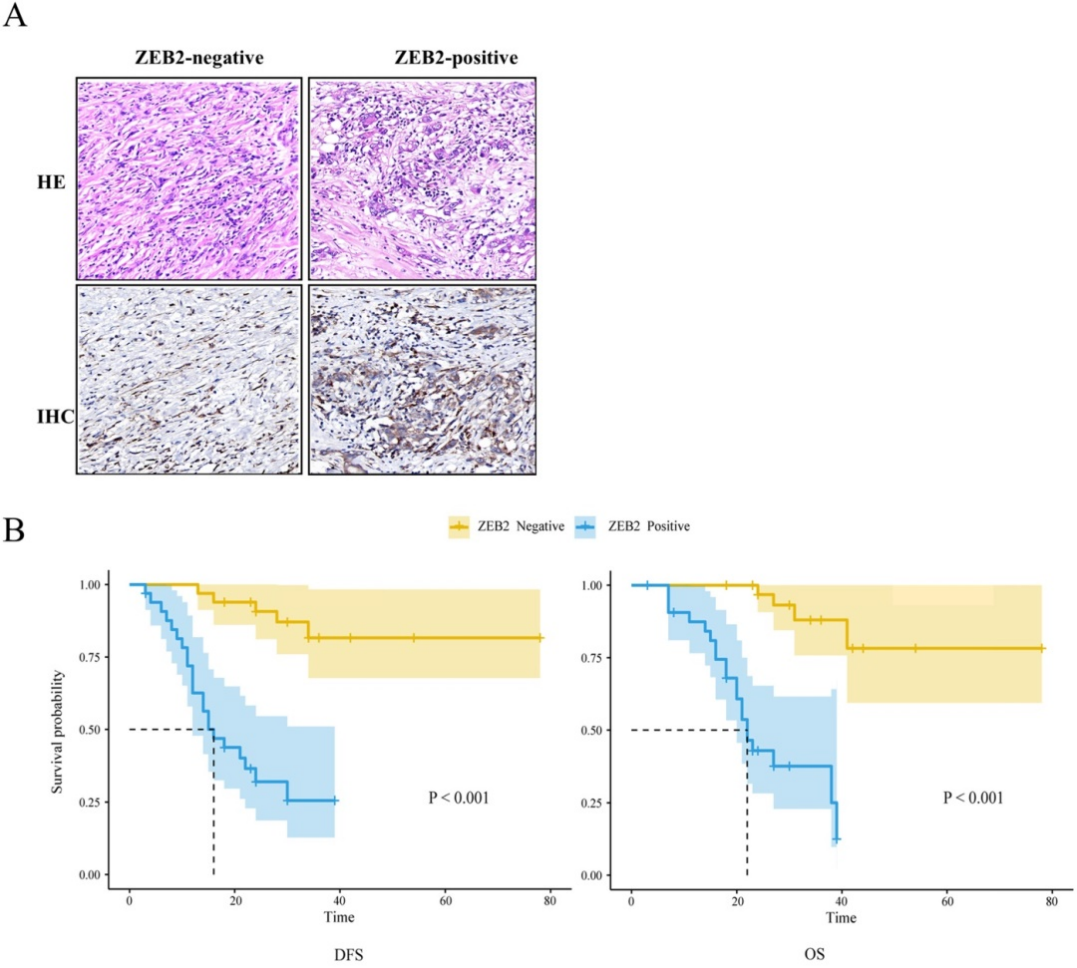
** The association between ZEB2 protein expression and the prognosis in 66 patients with gastric cancer who received cisplatin or oxaliplatin based adjuvant chemotherapy. (A)** ZEB2-negative and ZEB2-positive expression in postoperative tumor tissues of 66 GC patients who received cisplatin or oxaliplatin based adjuvant chemotherapy were detected using immunohistochemistry. HE, hematoxylin-eosin staining. IHC, immunohistochemistry. **(B)** Survival time of patients with gastric cancer. DFS and OS curves for ZEB2 expression. DFS, disease-free survival; OS, overall survival.

The Kaplan-Meier method and log-rank test were used to analyze the association between ZEB2 protein expression and the prognosis in 66 patients with gastric cancer who received cisplatin or oxaliplatin based adjuvant chemotherapy as shown in **Fig. 8**. The median DFS and median OS of patients with ZEB2 positive were 16 months and 22 months, respectively. However, the number of recurrence or death of patients with ZEB2 negative was small, the median DFS and median OS were not measured. The chi-square value between ZEB2 expression and the DFS was 26.5 (*P*<0.001). The chi-square value between ZEB2 expression and the OS was 28.4 (*P*<0.001). Statistical analyses demonstrated that ZEB2 expression was significantly associated with the overall survival and the disease-free survival of GC patients. The curves indicated that the DFS and OS of patients with positive ZEB2 expression are shorter than those of ZEB2-negative patients, thus ZEB2-positive patients benefit less from adjuvant chemotherapy with platinum-based drug.

## Discussion

Accumulating studies documented that the pathogenesis of GC is complicated involving several aspects. The interactions of genetic and environmental factors lead to a malignant phenotype 37. So far the cause of GC is still not entirely clear, we could not completely prevent it, thus the incidence of GC always keeps high. GC cases can be divided into early-stage and advanced-stage GC. Early-stage GCs are confined to the mucosa or submucosa, regardless of the presence of lymph node metastasis. The surgical operation is the first choice of the treatment for Early-stage GCs. For GC patients whose tumors cannot be resected surgically, chemotherapy is crucial to improve the survival rates and quality of life. Platinum, as one of the most important and widely used drugs in GC treatment, could promote apoptosis by damaging DNA, activating various signal transduction pathways 38. Unfortunately, platinum resistance is one of the major therapeutic challenges, thereby impacting its application and therapeutic efficacy. Therefore, there has been increasing interest in protect the effectiveness of platinum by minimizing the occurrence and impact of drug resistance. Although a large amount of studies displayed that changes in cellular uptake, decreased influx and increased drug efflux, mutation of target genes and enhancement of DNA repair activity are responsible for cisplatin resistance 6, 39, the mechanisms of cisplatin-induced resistance in GC still need to be further explored and validated. Therefore, the identification of novel tumor markers of GC is urgently needed to prompt the occurrence of tumors and predict the efficacy of cancer chemotherapy, ultimately helping to improve the efficiency of GC diagnosis.

miRNAs play a key role in human activities including tumour development by modulating gene expression. Dysregulated miRNAs in cancer are identified as tumor suppressor genes or oncogenes. Some miRNAs have reduced expression in cancer and leading to the overpression of their target oncogenes, suggesting that they may act as tumor suppressor genes. These miRNAs include miR-133b 40, let-7 41, miR-15 42, miR-34 [43]and so on. In contrast, a few miRNAs have increased expression in malignancies, giving rise to tumor formation. Known oncogenic miRNAs include miR-21 44, miR-155 45, miR-221 46 and so on. Intriguingly, for example, miR-125, can be either oncogenic or tumor suppressive, promoting or preventing the tumor growth at different stages 47. Moreover, miRNAs have been observed to be involved in tumor drug resistance. For instance, miR-138-5p possibly by targeting the DNA repair proteins ERCC1 and ERCC4 48, miR-135b by modulating expression of mammalian ste20-like kinase 1 (MST1) 49 and abnormal version of miR-34c were reported to effect cisplatin resistance in GC 50. To explore novel molecular marker of platinum resistance, we determined the differential miRNA and mRNA expression profiles among GC cells (cisplatin-resistant SGC7901/DDP cells vs. cisplatin-sensitive SGC7901 cells) by miRNA microarray analysis. A total of 48 miRNAs were found to be significantly differentially expressed (by >2-fold) in SGC7901/DDP cells relative to the parental cells. Among these, the most obvious expression difference was miR-338-5p. The expression of miR-338-5p in SGC7901/DDP cells was obviously lower than those in SGC7901 cells. Moreover, the differential expression of miR-338-5p was further confirmed by RT-qPCR analysis. More interestingly, miR-338-5p was generally low-expressed in various gastric cancer cell lines compared with normal gastric mucosal epithelial cells. Therefore miR-338-5p was selected as a further research target. *In vitro*, cisplatin-resistance experiments of GC cells confirmed that the overexpression of miR-338-5p could significantly reduce the cisplatin-resistance of SGC7901/DDP and BGC823 cells. More valuably, a significantly downregulated miR-338-5p expression level in cancer tissues compared to that in paracancer tissues for GC patients, and in the peripheral blood serum of GC patients compared with healthy adults. Furthermore, we analyzed the correlation between the expression of miR-338-5p and the pathological characteristics of GC patients. Results showed that the expression of miR-338-5p was lower in cancer tissues with TNM late stage than in cancer tissues with TNM early stage, in tissues with lymph node metastasis than in tissues with none lymph node metastasis. And compared with the tumors with less than 5 cm size, the tumors with more than 5 cm size have a lower expression level of miR-338-5p. And, in a very significant way, we found that patients with lower miR-338-5p expression in both cancer tissues and serum had generally poor survival through 5-year follow-up of 24 patients with GC. Therefore, the above data indicate that miR-338-5p may act as a tumor-suppressor gene in GC.

Generally, miRNAs mediate a series of biological processes through different target sites and their downstream target mRNAs function primarily depend on the specific 3'- untranslated region (3'-UTR) context 51. The DIANAmT, miRanda, miRDB, miRWalk, RNAhybrid, PICTAR4, PICTAR5, PITA, RNA22 and Targetscan databases predicted ZEB2 as a target of miR-338-5p. In the previous experiment, we also conducted mRNA microarray analysis between SGC7901/DDP cells and parental SGC7901 cells 35. Results showed that the expression of ZEB2 mRNA and protein in SGC7901/DDP cells were higher than that in SGC7901 cells.

ZEB2, as a key member of the Snail gene family, is closely associated with the biological processes of numerous tumors 29, 52. Additionally, it has been reported that ZEB2 plays a major role in EMT by combining the E-box sequence of E-cadherin and then suppressing the transcription of numerous genes. As a transcription factor that inhibits E-cadherin, ZEB2 was considered as a master EMT activator and was associated with the malignant phenotypes of cancers. Increasing studies also showed that ZEB2 was regulated by miRNAs in cancer. MiR-1179 targets ZEB2 to inhibit the growth and metastasis of hepatocellular carcinoma 53. MiR-377 was found to restrain aggressiveness and EMT via repression of ZEB2 in colon cancer 54. Therefore, ZEB2 may be a target of miR-338-5p. For the purpose of clarifying the potential relationship between miR-338-5p and ZEB2, We then transfected miR338-5p/mimics into SGC7901/DDP and BGC823 cells which originally has a lower expression of miR-338-5p.

ZEB2 was negatively regulated by miR-338-5p in GC cells. In addition, results of immunohistochemistry confirmed that a significantly upregulated ZEB2 expression level in tumor tissues compared to that in the adjacent para-tumor tissues. Furthermore, patients with large volume of tumor, lymph node metastasis and late TNM stage were associated with a marked higher expression level of ZEB2 on average, suggesting that high expression of ZEB2 may be associated with greater risk of poor prognosis. In fact, the survival prognosis of GC patients with ZEB2 positive expression tumor tissue was significantly worse than that of patients with ZEB2 negative tumor tissue. More importantly, the expression of miR-338-5p in tumor tissues and peripheral blood of GC patients with ZEB2 positive expression in tumor tissues was significantly lower than that of GC patients with ZEB2 negative expression in tumor tissues. In combination with cell experiments, ZEB2 expression in tumor tissues is negatively correlated with miR-338-5p expression in tumor tissues and peripheral blood of GC patients. In this study, we also conducted a retrospective study of patients who received platinum-containing chemotherapy clinically. GC patients with ZEB2-negative expression in tumor tissues were better in DFS and OS after receiving platinum-containing adjuvant chemotherapy than GC patients with ZEB2-positive expression, which indicated that ZEB2 expression was a negative factor in the efficacy of platinum-containing chemotherapy.

Although the mechanism of differential expression of miR-338-5p/ZEB2 in GC and the major signaling pathways involved are still unclear and more studies are needed, this study provides additional insight into the search for molecular targets that can be used for the diagnostic, prognostic and therapeutic predictive of platinum-based chemotherapy.

## Conclusion

miR-338-5p could reverse the cisplatin-resistance of GC cells, and miR-338-5p in cancer tissue and serum were closely related to the clinicopathological parameters and prognosis of GC, and exerts a tumor suppressor effect. ZEB2, which is negatively regulated by miR-338-5p, could increase the resistance of GC cells to cisplatin, and the expression of ZEB2 in cancer tissues is negatively correlated with miR-338-5p in cancer tissues and serum, thus playing the role of promoting tumor. GC patients with ZEB2 positive in tumor tissues have poor efficacy when receiving platinum-containing chemotherapy.

## Supplementary Material

Supplementary tables.

## Figures and Tables

**Table 1:** Clinical characteristics of GC patients and healthy adults

Characteristics	50 cases of GC patients		66 cases of GC patients received platinum-based adjuvant chemotherapy		30 cases of healthy adults	
	Cases, N	%	Cases, N	%	Cases, N	%
**Gender**						
Male	26	52	49	74	14	47
Female	24	48	17	26	16	53
**Age (years)**						
<55	14	28	26	39	9	30
≥55	36	72	40	61	21	70
**Tumor size (cm)**						
<5	35	70	38	58		
≥5	15	30	28	42		
**Differentiated degree**						
High/middle	28	56	31	47		
Low/un	22	44	35	53		
**Lymph node metastasis**						
Yes	30	60	50	76		
No	20	20	16	24		
**TNM stage**						
I/II	38	76	27	41		
III/IV	12	24	39	59		

**Table 2:** The IC50 of cisplatin in the GC cells (µg/mL, mean ±sd)

Cells	IC50
SGC7901/DDP	7.10±0.62
SGC7901/DDP-NC	7.18±0.69
SGC7901/DDP-miR-338-5p-mimics	3.75±0.15
SGC7901/DDP-ZEB2-KD	2.89±0.51
BGC823	2.18±0.09
BGC823-NC	2.27±0.41
BGC823-miR-338-5p-mimics	1.41±0.19
BGC823-ZEB2-KD	0.93±0.15

SGC7901/DDP-miR-338-5p-mimics, SGC7901/DDP cells transfected with microRNA-338-5p mimisc; SGC7901/DDP-ZEB2, SGC7901/DDP cells transfected with ZEB2-siRNA; BGC823-miR-338-5p-mimics, BGC823 cells transfected with microRNA-338-5p mimisc; BGC823-ZEB2, BGC823 cells transfected with ZEB2-siRNA.

**Table 3:** Expression of miR-338-5p in GC clinical specimens and the correlation with the clinicopathological features

Clinicopathological factors	Total	GC tissues relative to the para-tissues		Peripheral blood serum	
		miR-338-5p	P-value	miR-338-5p	P-value
**Gender**					
Male	26	1.40±1.82	0.332	1.05±1.54	0.919
Female	24	1.00±0.83		1.00±1.64	
**Age (years)**					
<55	14	0.95±0.93	0.889	1.29±2.01	0.581
≥55	36	1.00±0.86		1.00±1.53	
**Tumor size (cm)**					
<5	35	4.10±4.16	0.006	23.67±28.04	0.003
>5	15	1.00±0.37		1.00±0.86	
**Differentiated degree**					
High/middle	28	0.63±0.44	0.169	0.939±1.43	0.887
Low/un	22	1.00±1.31		1.00±1.58	
**Lymph node metastasis**					
Yes	30	0.45±0.85	0.017	0.17±0.35	<0.001
No	20	1.00±0.53		1.00±0.94	
**TNM stage**					
I/II	38	4.57±4.74	0.013	35.40±44.92	0.021
III /IV	12	1.00±0.31		1.00±0.83	
**ZEB2**					
Positive	36	0.34±0.23	<0.001	0.22±0.31	<0.001
Negative	14	1.00±0.98		1.00±0.99	

**Table 4:** MiR-338-5p target prediction

MicroRNA	Gene	DIANAmT	miRanda	miRDB	miRWalk	PICTAR5	Targetscan	SUM
miR-338-5p	ABCA8	1	1	1	1	1	1	6
miR-338-5p	PGM2L1	1	1	1	1	1	1	6
miR-338-5p	SNCA	1	1	1	1	1	1	6
miR-338-5p	TGFBR1	1	1	1	1	1	1	6
miR-338-5p	ZEB2	1	1	0	1	1	1	5
miR-338-5p	ATXN1	1	0	1	1	1	1	5
miR-338-5p	BMP2	1	1	0	1	1	1	5
miR-338-5p	CHL1	1	1	1	0	1	1	5
miR-338-5p	DNM3	0	1	1	1	1	1	5
miR-338-5p	DOCK4	1	1	0	1	1	1	5
miR-338-5p	FGF2	1	1	1	1	1	0	5
miR-338-5p	GCLC	1	1	0	1	1	1	5
miR-338-5p	GRB10	1	0	1	1	1	1	5
miR-338-5p	KAL1	1	1	1	0	1	1	5
miR-338-5p	PAPPA	1	1	0	1	1	1	5
miR-338-5p	TMEM2	1	1	0	1	1	1	5
miR-338-5p	SOX7	1	1	0	1	1	1	5

**Table 5:** Correlation analysis of miR-338-5p and mRNA

Source.GeneSymbol	Target	Correlation	P.value	Target.GeneSymbol
hsa-miR-338-5p	A_33_P3379886	-0.998464869	3.53E-06	FGF2
hsa-miR-338-5p	A_24_P294842	-0.998034103	5.79E-06	ATXN1
hsa-miR-338-5p	A_23_P122863	-0.997474742	9.56E-06	GRB10
hsa-miR-338-5p	A_23_P306987	-0.997283439	1.11E-05	SOX7
hsa-miR-338-5p	A_23_P371266	-0.996673459	1.66E-05	DNM3
hsa-miR-338-5p	A_33_P3342305	-0.996054667	2.33E-05	ABCA8
hsa-miR-338-5p	A_33_P3243832	-0.995107125	3.59E-05	ZEB2
hsa-miR-338-5p	A_33_P3363260	-0.995102907	3.59E-05	PGM2L1
hsa-miR-338-5p	A_23_P94552	-0.993036245	7.26E-05	TMEM2
hsa-miR-338-5p	A_23_P145114	-0.992956953	7.42E-05	GCLC
hsa-miR-338-5p	A_23_P212241	-0.99230844	8.85E-05	CHL1
hsa-miR-338-5p	A_33_P3237150	-0.992149001	9.22E-05	BMP2
hsa-miR-338-5p	A_23_P59637	-0.991899554	9.82E-05	DOCK4
hsa-miR-338-5p	A_33_P3331451	-0.991196753	0.000115905	TGFBR1
hsa-miR-338-5p	A_33_P3599591	-0.990872328	0.000124591	PAPPA

**Table 6:** Expression of ZEB2 protein in GC tumor and para-tumor tissues

	Total	ZEB2		Positive percent	χ^2^	P-value
		Positive	Negative			
Tumor tissues	50	36	14	72%	39.72	<0.001
Adjacent tissues	50	5	45	10%		

**Table 7:** Correlation between the expression of ZEB2 protein and the clinicopathological features of GC

Clinicopathological factors	Total	ZEB2		Positive percent	P-value
		Positive	Negative		
**Gender**					
Male	26	19	7	73.07%	0.86
Female	24	17	7	70.83%	
**Age (years)**					
<55	14	8	6	57.14%	0.15
≥55	36	28	8	77.77%	
**Tumor size (cm)**					
<5	35	22	13	62.85%	0.028
≥5	15	14	1	93.33%	
**Differentiated degree**					
High/middle	28	21	7	75%	0.59
Low/un	22	15	7	68.18%	
**Lymph node metastasis**					
Yes	30	27	3	90%	0.001
No	20	9	11	45%	
**TNM stage**					
I/II	38	23	15	60.52%	0.044
III/IV	12	11	1	91.66%	
