# Supplementary material for: miR-338-5p-ZEB2 axis in Diagnostic, Therapeutic Predictive and Prognostic Value of Gastric Cancer
Source: J Cancer. 2021 Sep 23;12(22):6756–72. doi: 10.7150/jca.58249 (PMC8518007; doi:10.7150/jca.58249)
Supplement: Supplementary file 1 — Supplementary tables. [file jcav12p6756s1.pdf]

Supplementary table 1. Patients with GC who underwent surgery in the prospective study.

Number	ZEB2 expression	miR-338-5p expression in seurm	miR-338-5p expression in tumor tissures	DFS		OS	
				Time (months)	State	Time (months)	State
1	Negative	1.000000000	0.870550563	52	Survive	52	Survive
2	Negative	2.297396710	0.574349177	50	Survive	50	Survive
3	Negative	2.639015822	0.353553391	51	Survive	51	Survive
4	Negative	2.828427125	0.406126198	52	Survive	52	Survive
5	Negative	4.000000000	1.071773463	52	Survive	52	Survive
6	Negative	6.964404506	2.143546925	52	Survive	52	Survive
7	Negative	1.414213562	0.466516496	52	Survive	52	Survive
8	Negative	0.933032992	0.466516496	52	Survive	52	Survive
9	Negative	0.143587294	0.116629124	5	Relapse	6	Death
10	Positive	0.203063099	0.153893052	50	Survive	50	Survive
11	Positive	0.615572207	0.500000000	46	Relapse	52	Survive
12	Positive	0.615572207	0.116629124	51	Survive	51	Survive
13	Positive	0.010308656	0.189464571	15	Relapse	23	Death
14	Positive	0.014578640	0.050765775	4	Relapse	5	Death
15	Positive	0.029157281	0.053893052	3	Relapse	5	Death
16	Positive	0.033492921	0.071793647	6	Relapse	12	Death
17	Positive	0.066985841	0.058314562	4	Relapse	6	Death
18	Positive	0.164938489	0.094732285	12	Relapse	24	Death
19	Positive	0.250000000	0.058314562	9	Relapse	24	Death
20	Positive	0.267943366	0.071793647	15	Relapse	24	Death
21	Positive	0.287174589	0.076946526	24	Relapse	40	Death
22	Positive	1.071773463	0.125000000	45	Relapse	73	Death
23	Positive	1.866065983	0.116629124	27	Relapse	75	Death
24	Positive	0.082469244	0.076946526	1	Relapse	1	Death

Supplementary table 2. Patients underwent 4 to 6 courses of adjuvant chemotherapy with cisplatin or oxaliplatin based drug in the retrospective studys.

Number	ZEB2 expression	DFS		OS	
		Time (months)	State	Time (months)	State
1	Negative	18	Survive	18	Survive
2	Positive	18	Survive	18	Survive
3	Positive	18	Survive	18	Survive
4	Negative	23	Survive	23	Survive
5	Positive	23	Survive	23	Survive
6	Positive	23	Survive	23	Survive
7	Negative	24	Survive	24	Survive
8	Negative	24	Survive	24	Survive
9	Positive	24	Survive	24	Survive
10	Negative	24	Survive	24	Survive
11	Positive	24	Survive	24	Survive
12	Negative	30	Survive	30	Survive
13	Negative	30	Survive	30	Survive
14	Negative	30	Survive	30	Survive
15	Positive	30	Survive	30	Survive
16	Negative	30	Survive	30	Survive
17	Negative	30	Survive	30	Survive
18	Positive	30	Survive	30	Survive
19	Negative	30	Survive	30	Survive
20	Positive	30	Survive	30	Survive
21	Negative	30	Survive	30	Survive
22	Negative	30	Survive	30	Survive
23	Negative	34	Survive	34	Survive
24	Negative	34	Survive	34	Survive
25	Negative	36	Survive	36	Survive
26	Negative	36	Survive	36	Survive
27	Negative	36	Survive	36	Survive
28	Negative	36	Survive	36	Survive
29	Negative	36	Survive	36	Survive
30	Negative	36	Survive	36	Survive
31	Positive	39	Survive	39	Survive
32	Negative	42	Survive	42	Survive
33	Negative	42	Survive	42	Survive
34	Negative	54	Survive	54	Survive
35	Negative	54	Survive	54	Survive

36	Negative	54	Survive	54	Survive
37	Negative	54	Survive	54	Survive
38	Negative	78	Survive	78	Survive
39	Positive	8	Relapse	8	Survive
40	Positive	3	Survive	3	Survive
41	Positive	12	Relapse	20	Death
42	Positive	22	Relapse	27	Survive
43	Negative	13	Relapse	27	Death
44	Positive	12	Relapse	20	Death
45	Positive	21	Relapse	27	Death
46	Positive	12	Relapse	18	Death
47	Negative	34	Relapse	44	Survive
48	Positive	6	Relapse	7	Death
49	Negative	28	Relapse	31	Death
50	Positive	11	Relapse	22	Death
51	Positive	3	Relapse	7	Death
52	Positive	14	Relapse	22	Death
53	Positive	7	Relapse	7	Death
54	Positive	11	Relapse	16	Death
55	Positive	18	Relapse	21	Death
56	Positive	15	Relapse	21	Death
57	Positive	9	Relapse	15	Death
58	Positive	10	Relapse	18	Death
59	Positive	16	Relapse	16	Death
60	Negative	24	Relapse	41	Death
61	Positive	15	Relapse	23	Death
62	Positive	14	Relapse	14	Death
63	Negative	16	Relapse	24	Death
64	Positive	30	Relapse	38	Death
65	Positive	24	Relapse	39	Death
66	Positive	4	Relapse	11	Death
